# Repeated Head Exposures to a 5G-3.5 GHz Signal Do Not Alter Behavior but Modify Intracortical Gene Expression in Adult Male Mice

**DOI:** 10.3390/ijms26062459

**Published:** 2025-03-10

**Authors:** Julie Lameth, Juliette Royer, Alexandra Martin, Corentine Marie, Délia Arnaud-Cormos, Philippe Lévêque, Roseline Poirier, Jean-Marc Edeline, Michel Mallat

**Affiliations:** 1 Institut du Cerveau, ICM, Inserm U 1127, CNRS UMR 7225, Sorbonne Université, 75013 Paris, France; 2 Institut des Neurosciences Paris-Saclay (NeuroPSI), CNRS, Université Paris-Saclay, 91400 Saclay, Franceroseline.poirier@universite-paris-saclay.fr (R.P.); jean-marc.edeline@universite-paris-saclay.fr (J.-M.E.); 3 XLIM, CNRS UMR 7252, 123 Avenue Albert Thomas, Université de Limoges, 87000 Limoges, France; 4 1 rue Descartes, Institut Universitaire de France (IUF), 75005 Paris, France

**Keywords:** 5G, electromagnetic field, memory, behavior, transcriptome, mitochondria, glutamatergic synapse

## Abstract

The fifth generation (5G) of mobile communications promotes human exposure to electromagnetic fields exploiting the 3.5 GHz frequency band. We analyzed behaviors, cognitive functions, and gene expression in mice submitted to asymmetrical head exposure to a 5G-modulated 3.5 GHz signal. The exposures were applied for 1 h daily, 5 days per week over a six-week period, at a specific absorption rate (SAR) averaging 0.19 W/kg over the brain. Locomotor activities in an open field, object location, and object recognition memories were assessed repeatedly after four weeks of exposure and did not reveal any significant effect on the locomotion/exploration, anxiety level, or memory processes. mRNA profiling was performed at the end of the exposure period in two symmetrical areas of the right and left cerebral cortex, in which the SAR values were 0.43 and 0.14 W/kg, respectively. We found significant changes in the expression of less than 1% of the expressed genes, with over-representations of genes related to glutamatergic synapses. The right cortical area differed from the left one by an over-representation of responsive genes encoded by the mitochondrial genome. Our data show that repeated head exposures to a 5G-3.5 GHz signal can trigger mild transcriptome alterations without changes in memory capacities or emotional state.

## 1. Introduction

Wireless communications generate worldwide exposure of people to radiofrequency (RF) electromagnetic fields (EMF), which have raised concerns and debates on the public health impact of these physical agents. The common use of mobile phones promotes head exposure of users, which has stimulated a wealth of investigation aiming at determining the possible effect of EMF, implemented by second, third, and fourth generations (Gs) of mobile or wireless (WIFI based on IEEE 802.11) communications, on the central nervous system (CNS). The influence on behavioral or cognitive functions was investigated in human subjects and animal models [1,2,3].

Among these, rodent models allow assessment of spontaneous behaviors and emotional states or cognitive capacities such as learning and memory and allow analysis of the cellular and molecular mechanisms responsible for behavioral modifications induced by environmental agents. Regarding the effects of RF-EMF on the learning abilities and spatial memory of rodents, published works show heterogeneous results, reporting either no effect or, in contrast, significant modifications of learning or memory abilities [3,4,5,6,7]. The diversity most likely reflects the influence of a combination of experimental parameters that vary across studies. Parameters related to exposure conditions include frequency and modulation of the signals, exposure mode limited to the head or extended to the whole body, its duration and periodicity, as well as the energy absorbed at the level of the brain or the whole body, which is quantified by the specific absorption rate (**SAR**) expressed in W/kg.

Although 2G, 3G, and 4G systems remain in use, the use of 5G mobile communications is expanding fast and was expected to reach 2.3 billion subscriptions by the end of 2024 (Ericson mobility report, 2024; https://www.ericsson.com/en/reports-and-papers/mobility-report (accessed on 20 February 2025)). Such 5G telecommunications systems exploit RF bands not used in previous systems at around 3.5 GHz or 26 GHz. In contrast to exposures to millimetric 26 GHz RF that are fully absorbed within 1–2 mm thick skin or underneath tissues, head exposure to 3.5 GHz RF results in energy transfer that can reach brain cells [8,9]. So far, the effect of 3.5 GHz RF exposure on the CNS remains little investigated. A recent electroencephalographic assessment of healthy human adults submitted to 17 min of far-field exposure to a 5G-3.5 GHz signal revealed no significant overall changes in the electrical brain activity, and the brain-averaged SAR was estimated at a value as low as 0.008 mW/kg. However, alterations in power spectral densities occurred at the level of some electrodes during the exposure or within a 17 min time period following exposure, which depended on open or closed eye conditions [8]. No clear changes in the spontaneous electrophysiological activity of neuronal networks could be observed when using primary cultures of embryonic mouse neurons that were exposed to a 5G-3.5GHz signal applied for 15 min at SAR levels of 1 W/kg or 3 W/kg [10]. In addition to studies focused on neuronal activities, the behavioral and molecular consequences of 3.5 GHz RF were investigated in different animal models. Exposures of adult male guinea pigs applied for three days at whole-body SAR values of 2, 4, or 10 W/kg did not alter locomotor activities or hearing thresholds but triggered molecular and ultrastructural marks of oxidative stress at the level of the auditory cortex [11]. Short-term exposures with SAR value estimations ranging from 0.0026 to 0.26 W/Kg were found to increase activity and reduce sleep duration in male flies, whereas parental and embryonic exposures had opposite behavioral effects in F1 male offspring. Behavioral changes were associated with the altered expression of genes encoding heat shock proteins or circadian clock components and modifications in the levels of excitatory and inhibitory neurotransmitters [12]. Parental and embryonic exposures of flies also resulted in shortening of the mean development time, together with increased activity of antioxidant enzymes and modifications of the fly microbiota assessed at late larval stages [13]. Developmental impacts were also documented in zebrafish, showing that exposures during embryonic life can result in hypoactivity or anxiety-related behaviors at larval or adult stages [14,15,16]. Transcriptome profiling of zebrafish embryos performed at the end of the RF exposure revealed significant changes in transcript levels limited to 28 genes involved in metabolic pathways [15].

In the current study, we set up an experimental mouse model to evaluate whether one month of chronic exposure to a 5G-3.5 GHz signal (1 h/day; 5 days a week) in adulthood can alter exploration, anxiety, memory, and intracerebral gene expressions. The exposures were performed in awake, head-restrained animals to ensure daily head exposure at a power set to limit upper SAR values at around 0.5 W/kg within the CNS. After 5G exposure, the locomotor activities in an open field (OF) and learning and memory abilities of mice tested in novel object recognition and object location tasks were compared to those of pseudo-exposed mice, which were also awake and head-restrained, at the same time as the exposed mice, but the 5G-3.5 GHz signal was turned off. Unrestrained cage control mice were also used to evaluate the potential deficit induced by the restrained conditions. After completion of the behavioral task, the brains were quickly removed from the skull, and whole-genome mRNA profiling was performed in symmetrical areas of the right and left cerebral cortex, which differed owing to the local energy deposit, as indicated by our dosimetric analysis.

## 2. Results

### 2.1. Development of a Calibrated Head Exposure System for Awake Mice

**Figure 1:**
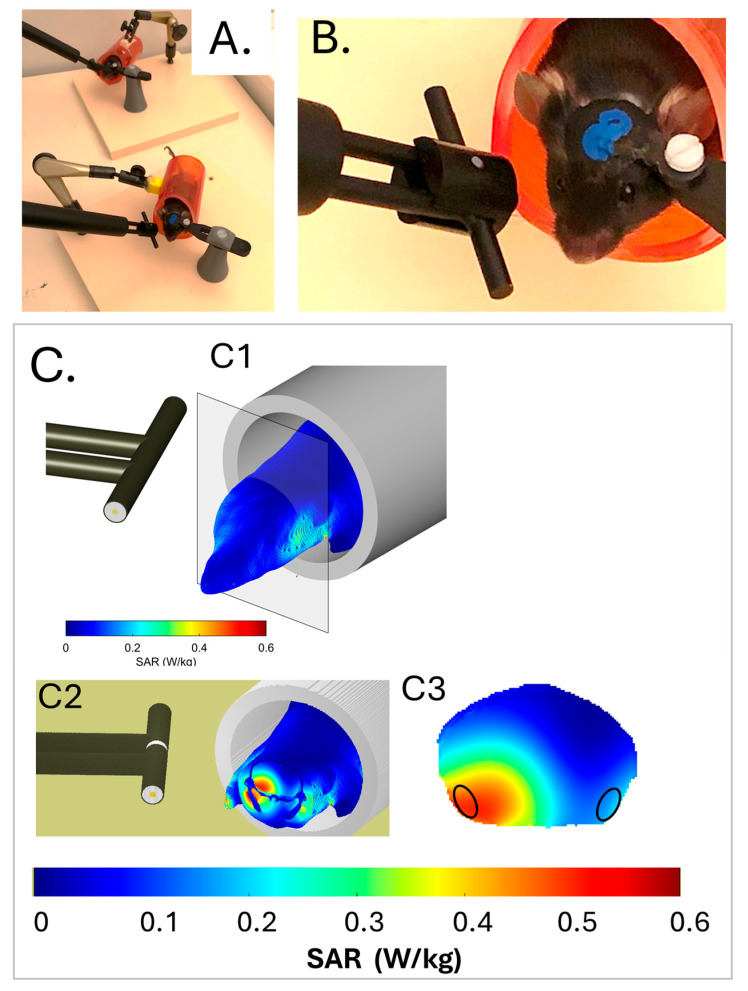
Exposure setup and dosimetry. (**A**,**B**) Head-restrained mice in red plastic tubes with dipole antennas positioned at the vicinity of the right temporal cortex. The low magnification view (**A**) shows two matched 5G-exposed and pseudo-exposed animals (the antenna of the pseudo-exposed mouse is not connected to the 5G-3.5 GHz generator). (**C**) Dosimetric analysis of specific absorption rates (SARs) in the mouse head and trunk. A heterogeneous model of the mouse and dipole antenna was used to evaluate the local SARs in the head and trunk at a spatial resolution of 0.2 mm^3^. (**C1**) Three-dimensional view illustrating SAR values at the surface of the head and trunk. (**C2**) Three-dimensional view with a coronal section showing external and internal distributions of the SAR values in the head. The area of the sectioned brain is delimited by a black contour. (**C3**) Two-dimensional view focused on the coronal brain sections. Areas of the right and left entorhinal-piriform cortex, which were sampled for transcriptomic analyses, are encircled. Bars show the color-code scale of SAR values.

Adult male mice (*n* = 32) were submitted to surgical implantation of a cranial hook, allowing fixed and reproducible positioning of the right side of their head close to an antenna emitting, or not, a 5G-modulated 3.5GHz signal (Figure 1A,B). Hook-implanted mice were either exposed to the 5G-3.5GHz signal (*n* = 12 animals) or pseudo (PSD)–exposed (5G signal generator switched off; *n* = 12 animals) for 1 h daily, 5 days a week. The 5G and PSD exposures were carried out with awake mice that were previously habituated to the head-restrained conditions. A third group of control (CTRL) mice (*n* = 8) were kept free of head movement restriction.

The distribution of energy in mice exposed to the 5G-3.5GHz signal was determined numerically using a numerical mouse model. Figure 1C shows the SAR distribution in the mouse head and anterior trunk of the mouse model, which matched that of the mice in terms of weight and size. Intracerebral SAR values (Figure 1(C3)) were the highest in ventral cortical areas ipsilateral to the antenna, which covered adjacent areas of the right entorhinal and piriform cortex (Ent-Pir Cx), reaching values of 0.43 ± 0.12 W/kg (volume-averaged value ± SD among the SAR voxel values over the defined volume). SAR values in the contralateral (left) Ent-Pir Cx dropped to 0.14 ± 0.05 W/kg. The whole brain-averaged SAR was 0.19 ± 0.12 W/kg. As the weight of the exposed mice was homogenous, differences in tissue thickness at the level of the head were probably negligible, so the actual SAR in the cerebral cortex was very similar from one exposed animal to another.

### 2.2. Assessing How Chronic 5G Exposure Affects Locomotion, Anxiety Level, or Memory

After 20 daily sessions of 1h-exposure, we assessed the effects of the 5G-3.5 GHz signal on several behavioral parameters and memory processes for six additional days. During this period, the daily 5G/pseudo-exposure sessions continued. Mice were behaviorally tested after 10 min of rest in their home/personal cage. Because head-fixed conditions could also induce behavioral alterations such as hyperactivity or increased anxiety, we also compared the performances of 5G-exposed and PSD-exposed mice with CTRL mice (without restraint) for these parameters.

**Figure 2:**
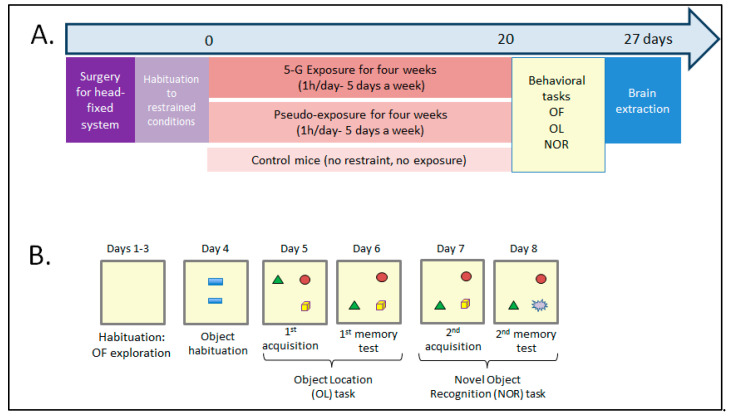
Timeline of the experimental procedures. (**A**) After surgery and habituation to restrained conditions, mice were exposed to a 5G-3.5 GHz signal for 1 h per day, 5 days a week over 4 weeks. After day 20, mice were submitted to behavioral tasks after each 1h-exposure session. As controls, the behavior of a group of mice kept under restraint conditions during the same period (pseudo-exposed group) and a group of control mice without exposure or restraint was also assessed. At the end, the brains of 5G- and pseudo-exposed mice were collected for biochemical analysis. (**B**) Behavioral tasks were used to assess exploration/locomotion in an open field (OF), and object recognition memory with object location (OL) and novel object recognition (NOR) tasks. Mice were allowed to explore the OF during three sessions (days 1–3). On day 4, they could explore two objects placed in the OF. From day 5, they were submitted to memory tasks. For the OL task, one object was displaced (here, the green triangle). For the NOR task, an object was replaced with a new one. In both conditions, because mice are spontaneously attracted by novelty, their recognition memory is assessed. Two 5G-exposed and one PSD-exposed animal had to be retired from analysis because their plastic hook was damaged during the last sessions: one before the behavioral tasks, one before the object habituation session, and one before the OL task. Therefore, the results presented below are from 8 CTRL mice, 10/11 5G-exposed mice, and 11 PSD-exposed mice.

Figure 2 illustrates the experimental procedures and their timelines. Each mouse underwent three sessions of exploration/locomotion in an open field (OF) (object habituation day 4), which were followed by one session in which the animals could explore two identical objects placed in the OF. The animals were then assessed for object recognition memory with object location (OL) and novel object recognition (NOR) tasks.

### 2.3. Chronic 5G Exposure Does Not Lead to Hyperactivity and Abnormal Anxiety

**Figure 3:**
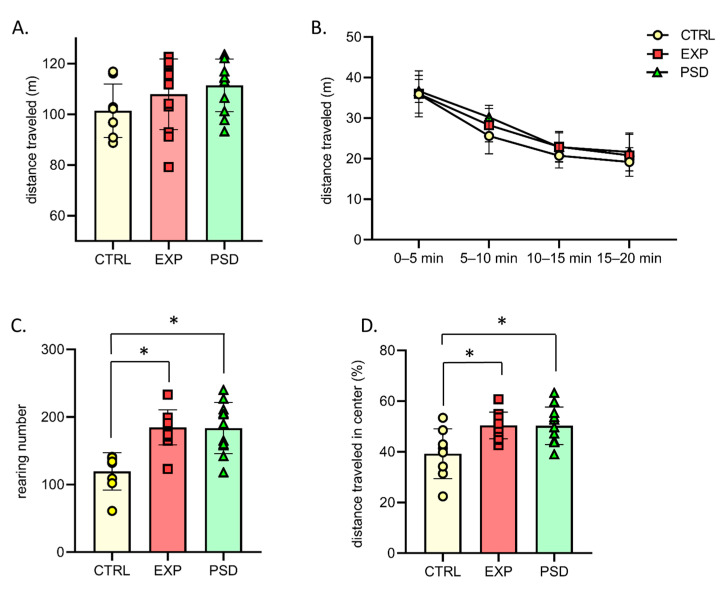
Behavioral parameters observed during the first 20 min session of the open field for CTRL (n = 8), EXP (5G-exposed, n = 12), and PSD (n = 11) mice. (**A**) Distance traveled in the open field during a 20 min session. One-way ANOVA, ns. (**B**) Evolution of distance traveled in the open field during the 20 min of the test. Two-way ANOVA between groups: ns groups × time session: ns. (**C**) Mean number of rearings during the 20 min session. One-way ANOVA, *p* < 0.05- Bonferroni’s multiple comparison; CTRL vs. EXP *p* < 0.05; CTRL vs. PSD: *p* < 0.05; EXP vs. PSD: ns. (**D**) Percentage of the distance traveled in the central area of the open field. One-way ANOVA, *p* < 0.05- Bonferroni’s multiple comparison; CTRL vs. EXP *p* < 0.05; CTRL vs. PSD: *p* < 0.05; EXP vs. PSD: ns. CTRL: control mice; EXP: 5G-exposed mice; PSD: pseudo-exposed mice. Errors bars represent ± SEM. * indicates a significant difference between groups of mice (*p* < 0.05). ns: non-significant.

During the first session in the OF, 5G-exposed (EXP) mice showed normal locomotion and exploration activity, and the total distance traveled was similar to those of PSD-exposed and CTRL mice (Figure 3A; one-way ANOVA, F(2,28) = 1.64; *p* = 0.21). Over the 20 min of the test, the distance traveled progressively decreased, revealing normal habituation to this novel environment, and no difference between groups was found (Figure 3B; Two-way ANOVA, 5 min session effect: F(3,84) = 134; *p* < 0.0001; group effect: F(2,28) = 1.64; *p* = 0.21, 5 min session × group: F(6,84) = 0.63; *p* = 0.7). Similar results were observed during the second and third sessions of habituation in the OF, with no difference between groups (Supplementary Table S1). Regarding another index of exploratory behavior, our analyses revealed that the numbers of rearings of 5G- and PSD-exposed mice were significantly increased compared to CTRL mice during the first 20 min session in the OF (Figure 3C; F(2,28) = 12.72; *p* = 0.0001; Bonferroni’s multiple comparison test: CTRL vs. EXP: *p* = 0.0003; CTRL vs. PSD: *p* = 0.0004). No difference was found between 5G- and PSD-exposed mice (*p* > 0.99). This increase in the number of rearings could be due to daily manipulation of the 5G-exposed and PSD-exposed mice in contrast to the CTRL mice. This interpretation is reinforced by the data observed during the second and third sessions, where the numbers of rearings were similar between the three groups (Supplementary Table S1). Finally, to assess whether 5G exposure could induce anxiety-like behaviors, we analyzed the percentage of the distance traveled in the central zone of the OF. For this parameter, the 5G- and PSD-exposed mice traveled similar distances in the central area of the OF. However, as shown in Figure 3D, the CTRL mice traveled significantly less distance in this central area compared to the exposed mice (Figure 3D for the first session, F(2,28) = 6.65; *p* = 0.0043; Bonferroni’s multiple comparison test: EXP vs. PSD: *p* > 0.99; CTRL vs. EXP: *p* = 0.0078; CTRL vs. PSD: *p* = 0.01). From the second session in the OF, there was no difference for this parameter between the three groups (Supplementary Data for the second and third sessions, Table S1), confirming that, during the first session in the OF, CTRL mice were slightly more anxious than 5G- and PSD-exposed, daily manipulated mice. We observed similar results when two objects were introduced into the OF for the first time. During this session (day 4), there was no difference in the time spent exploring the objects between 5G- and PSD-exposed mice, but CTRL mice spent significantly less time exploring the two objects than exposed mice (Supplementary Figure S1A: F(2,27) = 6.059; *p* = 0.0067; Bonferroni’s multiple comparison test: EXP vs. PSD: *p* > 0.99; CTRL vs. EXP: *p* = 0.01; CTRL vs. PSD: *p* = 0.017). Together, our analyses suggest that 5G-exposed mice did not display hyperactivity or abnormal anxiety, and their levels of exploration of the OF/objects were similar to the PSD-exposed mice.

### 2.4. Chronic 5G Exposure Does Not Alter Object/Object Location Recognition Memory

**Figure 4:**
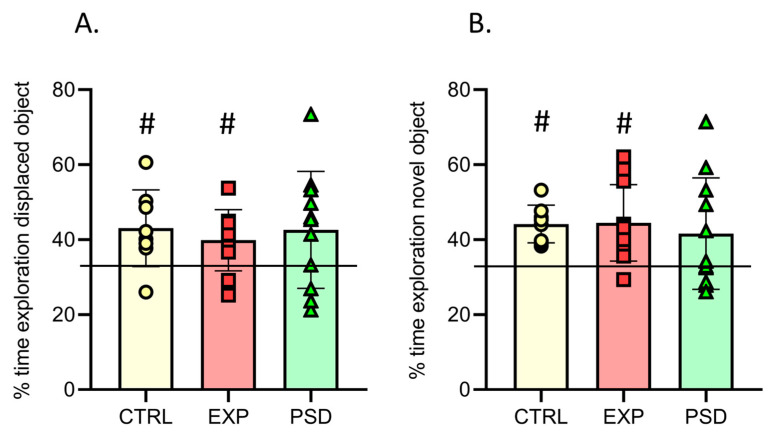
Memory tests. (**A**) Percentage of time spent exploring the displaced object during a 5 min test session in CTRL (n = 8), EXP (n = 10), and PSD (n = 11) mice. (**B**) Percentage of time spent exploring the novel object during a 5 min test session in CTRL (n = 8), EXP (n = 11), and PSD (n = 11) mice. Values are means ± SEM. The horizontal line at 33.33% indicates equal exploration of the displaced/novel and not displaced/familiar objects. (**A**,**B**) One-way ANOVA between groups, ns. For each group, one sample *t*-test compared to 33.33% (chance). # *p* < 0.05 compared to 33.33%. CTRL: control mice; EXP: 5G-exposed mice; PSD: pseudo-exposed mice.

To assess long-term memory in the OL and NOR tasks, the mice were first submitted to sessions of acquisition allowing them to explore three different objects for 15 min (Figure 2B, days 5 and 7). During these acquisition sessions, the CTRL, 5G, and PSD-exposed mice showed similar object exploration during the 15 min sessions (Supplementary Figure S1B,C, time to explore the three objects during the acquisition for OL: F(2,26) = 0.20; *p* = 0.81; for NOR: F(2,27) = 0.03; *p* = 0.96). Memory tests were performed 24 h after acquisition by moving one of the three objects (OL task) or replacing one familiar object with a novel object (NOR task, Figure 2B). Because mice are spontaneously attracted by novelty, we analyzed the time spent exploring the displaced object for the OL task and the novel object for the NOR task. As shown in Figure 4, 5G-exposed-mice showed preferential exploration of the displaced/novel object compared with not displaced/familiar objects (OL task: t = 2.533; *p* = 0.032; NOR task: t = 3.63; *p* = 0.0046), and their times exploring the displaced/novel object were similar to those of the control and PSD-exposed mice (OL: F(2,26) = 0.1985; *p* = 0.8212; NOR: F(2,27) = 0.2080; *p* = 0.8135). These findings demonstrate that the long-term object recognition memory and long-term memory of the spatial location of objects were not affected by the 27 h of chronic 5G exposure.

### 2.5. Effects of Chronic 5G Exposure on Gene Expression in the Cerebral Cortex

#### 2.5.1. Chronic 5G Exposure Differentially Affects Gene Expression in the Right and Left Ent-Pir Cx

To determine the effect of chronic 5G exposure on gene expression in the cerebral cortex, we analyzed the coding transcriptome in two symmetrical ventral brain regions, the right and left Ent-Pir Cx (Figure 1(C3)), in which the mean SAR levels were 0.43 W/kg and 0.14 W/kg, respectively. mRNA sequencing was carried out using tissue collected from 5G-exposed (n = 7) and pseudo-exposed (n = 8) animals that had been submitted to 27 h of exposure or PSD exposure over 6 weeks (see protocol in Figure 2). Changes in gene expression were analyzed by comparing the levels of transcripts (i) between the right and left Ent-Pir Cx in PSD-exposed mice, (ii) in the right Ent-Pir Cx between 5G-exposed and PSD-exposed animals, and (iii) in the left Ent-Pir Cx between 5G-exposed and PSD-exposed animals. Differences in the levels of transcripts were analyzed by calculating multiple hypothesis adjusted *p*-values using the Benjamini–Hochberg procedure to control the false discovery rate (FDR). They were considered statistically significant for FDR-adjusted *p*-values ≤ 0.05. Differentially expressed genes (DEG) were determined by FDR-adjusted *p*-values ≤ 0.05 and a fold-change (FC) threshold set to 1.2.

**Figure 5:**
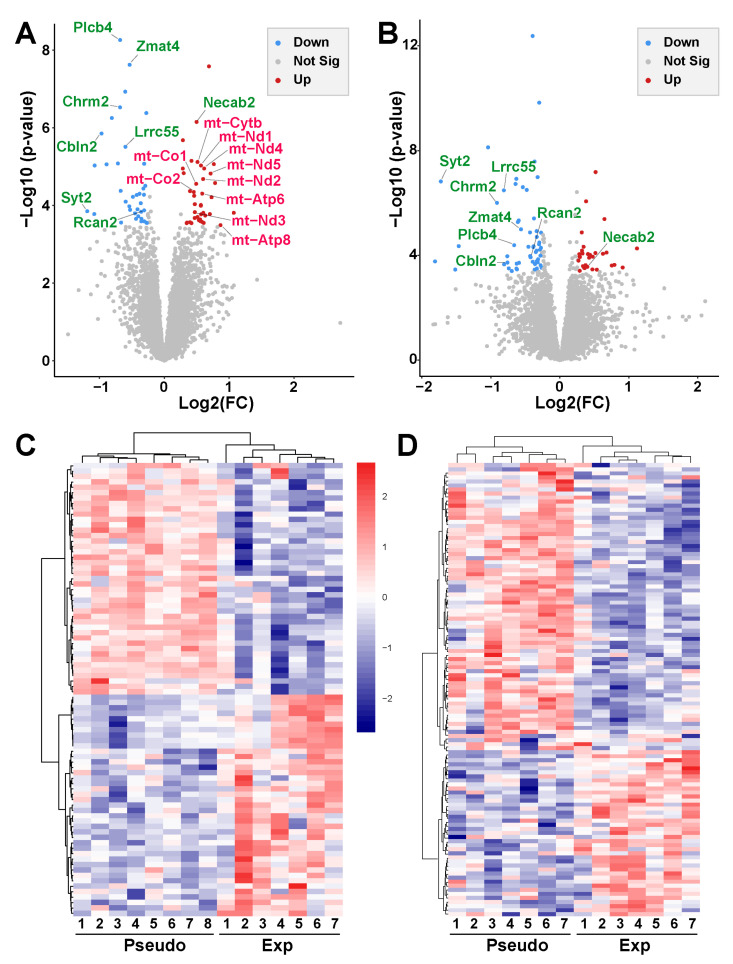
RNA-seq analysis of the Ent-Pir Cx. (**A**,**B**) Volcano plots of differential gene expression between 5G-exposed and pseudo-exposed animals (FC cutoff 1.2) in the right (**A**) and left (**B**) Ent-Pir Cx. The ordinates correspond to *p*-values adjusted with the Benjamini–Hochberg procedure (FDR-adjusted *p*-values). The genes that were significantly up-regulated or down-regulated in response to the 5G exposure are indicated by red or blue dots, respectively. Gray dots correspond to genes that were not differentially expressed. Up-regulated genes encoded by the mitochondrial (mt) genome are written in red. Up- or down-regulated genes common to the right and left Ent-Pir Cx are written in green. (**C**,**D**) Heat maps showing color-coded normalized expression levels of the genes, which were significantly modulated by the 5G exposure (FDR adjusted *p*-value ≤ 0.05) in the right (**C**) or left (**D**) Ent-Pir Cx. Gene clustering was performed using the Euclidean distance and ward agglomeration method. Pseudo (1–8) correspond to each of the pseudo-exposed mice; Exp (1–7) indicate 5G-exposed mice.

In PSD-exposed animals, as expected, the transcriptome profiles in the right and left Ent-Pir Cx were almost identical. Significant differences in the levels of transcripts (FC > 1.2, FDR-adjusted *p* < 0.05) between the right and left Ent-Pir Cx in pseudo-exposed mice were restricted to five genes (Supplementary Table S2) over a total of 12,423 genes, in which expression could be detected in these brain regions. A comparison of 5G-exposed and PSD-exposed animals revealed significant gene modulations triggered by the 5G-3.5 GHz signal. In the right Ent-Pir Cx, changes in the level of expression (FC > 1.2, FDR-adjusted *p* < 0.05) were limited to 77 genes, e.g., less than 0.7% of the expressed genes in this CNS region. These DEG comprised 40 up-regulated and 37 down-regulated genes in response to the chronic 5G exposures (Figure 5A; Supplementary Table S3).

In the left Ent-Pir Cx, the total number of DEG was slightly higher (84 genes), despite a lower SAR level compared with the right Ent-Pir Cx. These 84 DEG segregated into 30 up-regulated genes and 54 down-regulated genes in response to the 5G exposure (Figure 5B; Supplementary Table S4). Unsupervised clustering showed clear segregation between 5G-exposed and PSD-exposed animals based on significant differences in gene expression (Figure 5C,D). Of note, there was no effect of the 5G exposure on the five genes whose expressions differed between the right and left Ent-Pir Cx in PSD-exposed animals. Strikingly, the identity of the DEG markedly differed between the right and left Ent-Pir Cx, which only shared eight of their DEG (Figure 5A,B), seven of which were down-regulated both in the right and left Ent-Pir Cx.

#### 2.5.2. Chronic 5G Exposure Affects Genes Encoding Mitochondrial or Synapse Components

To specify biological processes, cellular components, or molecular functions potentially affected by the 5G-triggered gene modulation, we performed gene ontology (GO)-based enrichment analysis of the DEG.

**Figure 6:**
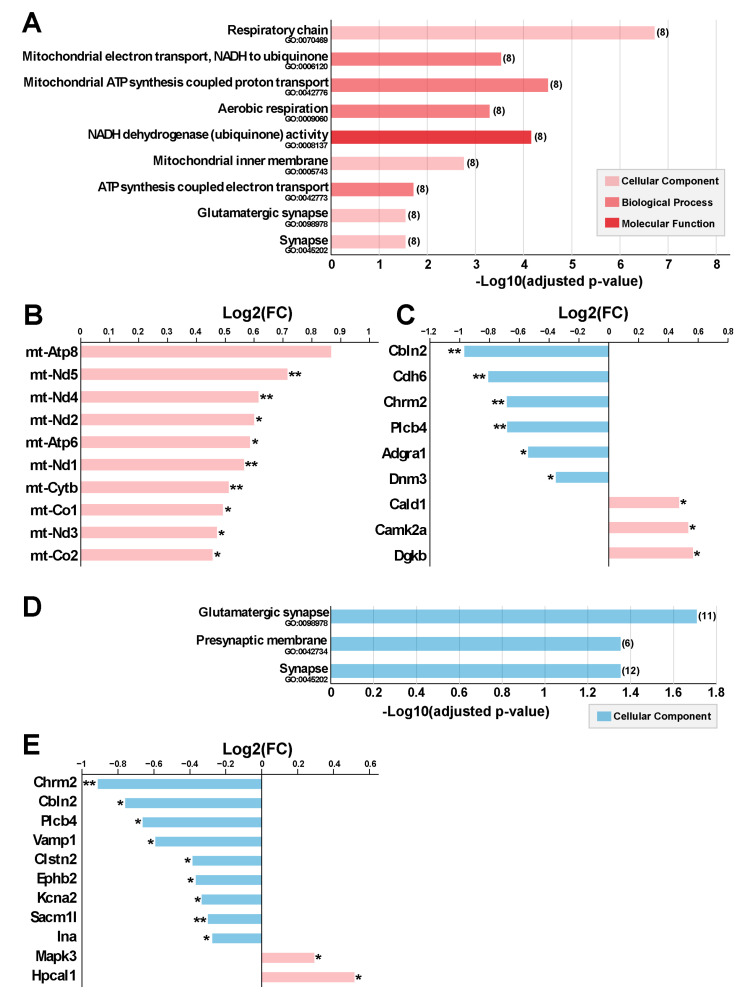
Gene ontology (GO) annotation of the differentially expressed genes (DEG) between 5G-exposed and pseudo-exposed mice. (**A**–**C**) Right Ent-Pir Cx. (**A**) Significantly enriched GO terms and enrichment *p*-values are indicated (Benjamini–Hochberg procedure). The number of DEG related to the GO terms is in brackets. (**B**,**C**) DEG associated with mitochondria-related GO terms (**B**) or the “glutamatergic synapse” term (**C**). DEG names, fold changes (Log2(FC)) and significancy of the modulations (* FDR-adjusted *p*-value < 0.05; ** < 0.01) are indicated. (**D**,**E**) Left Ent-Pir Cx. (**D**) Enriched terms and *p*-values. (**E**) DEG associated with the glutamatergic synapse GO term.

For the right Ent-Pir Cx, significant enrichments in GO terms were mostly related to the mitochondrial oxidative phosphorylation system (OXPHOS) [17] (Figure 6A). The enrichment in mitochondrial terms corresponded to an over-representation of genes of the mitochondrial (mt) genome among the DEG (Figure 5A). The mt genome contains 13 genes encoding protein subunits of the ATP-producing OXPHOS [18]. Ten of these mt genes were up-regulated (1.38 < FC < 1.83) upon exposure to the 5G signal (Figure 6B). These genes encode core subunits of enzyme complexes I, III, IV, and V of the OXPHOS (Supplementary Table S3), which are embedded in or anchored to the inner mitochondrial membrane [19]. Three of the mt genes encoding peptides (mt-CO3, mt-ND4L and mt-ND6) were not identified as DEG, given our stringent criterion for statistical significance, which set the threshold for FDR-adjusted *p*-values to 0.05. However, the mt-CO3 (FC = 1.35; FDR-adjusted *p* = 0.059), mt-ND4L (FC = 1.48; FDR-adjusted *p* = 0.122), and mt-ND6 (FC = 1.46; FDR-adjusted *p* = 0.142) genes showed a clear trend toward up-regulation in the right Ent-Pir Cx of 5G-exposed animals.

In both the right and left Ent-Pir Cx, GO-based analyses highlighted significantly enriched terms defining cellular components involved in neurotransmission, such as synapses or glutamatergic synapses (Figure 6A,D). These enrichments arose predominantly from down-regulation but also from up-regulation of genes (Figure 6C,E). In the right and left Ent-Pir Cx, 9 and 11 DEG encoded components of glutamatergic synapses, respectively. However, only three of these DEG (Chrm2, Plcb4, and Cbln2) were common to the right and left Ent-Pir Cx (Figure 6C,E).

Together, our results show that chronic head exposure to the 5G-3.5 GHz signal leads to altered expression of genes in the cerebral cortex. The identities of the DEG markedly vary when comparing two homologous cortical regions that differ owing to the local SAR values during the 27 h of exposure.

## 3. Discussion

The recent launch and expansion of 5G telecommunication systems require assessment of the brain impact of the EMF generated by this technology. We have set up an asymmetrical head-exposure system to a calibrated 5G-modulated 3.5 GHz signal, which was adapted to awake adult mice. Dosimetric analyses indicate that the average brain SAR level was 0.19 W/kg, whereas maximal SAR levels in the range of 0.5 W/kg were reached in the right Ent-Pir Cx. We investigated the effect of repeated head exposures on the mouse CNS through assessment of animal behaviors and transcriptome profiling. Our results show no significant effect of the 5G signal on locomotion, anxiety level, or memory capacities, but they reveal biological responses quantified by modulations of gene expression in the cerebral cortex.

At the behavioral level, we analyzed the influence of a 5G-3.5 GHz signal on mice, assessing locomotion and exploration in an OF. None of the analyzed behavioral parameters, such as distance traveled, number of rearings, or distance in the central area, revealed any significant alteration in exploration and anxiety levels when applying the 5G signal for 1 h daily, 5 days a week over a six-week period. Similarly, we found no significant effect of the 5G signal on memory abilities, as determined in object location and novel object recognition tasks. Our results differ from those of a recent study showing that 1 h daily of whole-body exposure of adult mice to pulsed 3.5 GHz RF carried out over 35 days led to increased anxiety levels, albeit without modification of learning or memory performances [20]. However, this report did not specify the pulse mode that was applied to the exposures nor the SAR levels that were reached in the brain or the body of the exposed mice. So far, to our knowledge, no other rodent study has investigated how chronic exposure to 3.5 GHz RF during adulthood might affect emotional states or memory capacities. However, increases in anxiety levels were detected in adult zebrafish that were exposed to 3.5 GHz for 42 h at a high SAR level (8.7 W/kg) during embryonic stages of life, as indicated by an enhanced escape response to predators [15]. Zebrafish embryos exposed for 1 or 4 h to 3.5 GHz RF at an estimated SAR of 1.1 W/kg subsequently displayed anxiety-like behavior during the larval stage of life, which was marked by modifications in the thigmotaxis or wall-hugging behavior triggered by visual or auditory stimuli [16]. Behavioral impacts of RF-EMF depend on various parameters, including not only the number, duration, and power of the exposures but also the type of modulation applied to the emitted RF, which changes according to the generation of mobile communication systems [3,21,22]. Our study provides a first analysis of the effect of a 5G-modulated 3.5 GHz signal on anxiety or memory capacities in mammals.

Transcriptome profiling was previously used to determine gene responses in the brains of rodents that were exposed to GSM-related EMF [23,24,25,26,27,28]. Here, we found that chronic 5G-3.5 GHz exposure triggered significant modulations in less than 1% of the expressed genes in the right Ent-Pir Cx, where the local SAR level reached its maximal levels. As expected, in the absence of exposure to EMF, the right and left Ent-Pir Cx displayed virtually identical transcriptome profiles (differences in gene expression limited to five over more than 12,000 expressed genes in pseudo-exposed animals). Due to the asymmetrical head exposure, we compared the gene responses in the right and left Ent-Pir Cx, which were differentially exposed to the 5G-3.5 GHz signal. We observed that a three-fold decrease in the mean SAR level was associated with marked changes in the identity of the DEG without a reduction in the number of responsive genes. In fact, the number of DEG was higher in the left Ent-Pir Cx (84 DEG, mean local SAR of 0.14 W/kg) than in the right Ent-Pir Cx (77 DEG, mean SAR: 0.43 W/kg), whereas only eight DEG were shared by the right and left cortical areas. This result indicates that the magnitude of the gene response does not continuously increase with the level of energy absorbed in the tissue when SAR values range between 0.1 and 0.5 W/kg. EEG studies performed with human subjects indicate that visual activities impact the cortical cell responses triggered by low-power head exposure to a 5G-3.5 GHz signal [8]. We speculate that differences in SAR values and neuronal activities concurred in shaping different gene responses in the right and left Ent-Pir Cx.

Despite the marked differences in gene identities, the profiles of DEG in the right and left Ent-Pir Cx shared significant enrichments in genes related to glutamatergic synapses that convey defined excitatory neurotransmissions in these cortical areas [29]. The entorhinal cortex is the primary spatial input to the hippocampus and bridges neuronal signaling between the hippocampus and cortical regions, including the piriform cortex. Neuronal activity in the entorhinal cortex plays a major role in spatial memory and navigation (reviewed in [19,30]) and was also proposed to be involved in object recognition memory [31]. Our current behavioral analyses show no alteration of these memory capacities in the animals exposed to the 5G-3.5GHz signal. However, this does not discard the possibility that the synapse-related DEG responses reflected alterations in excitatory neurotransmission, which remained too weak to affect memory performances. In line with this hypothesis, altered neuronal activity-sparing cognition was recently documented in a study combining behavior assessment and intracerebral cFos imaging in rats submitted to subchronic exposure to a GSM-900 MHz signal (45 min exposure daily over two weeks at a brain averaged SAR of 1 or 3.5 W/kg). The GSM exposures led to reduced neuronal activity in cortical and hippocampal areas involved in memory tasks without modification of memory performance [32]. Furthermore, the suppressive effects of RF-EMF on glutamatergic neurotransmission or neuronal bursting activity were observed in culture studies in which rat cortical or hippocampal neurons were exposed to GSM-1800 MHz microwaves at SAR levels ≥ 0.1 W/kg [33,34] as well as in vivo in rats submitted to acute head exposures to GSM-900, 1800 MHz, or LTE-1800 MHz signals [35,36,37,38].

While our study highlights synapse-related DEG in both the right and left Ent-Pir Cx, we found that profiles of DEG in the right Ent-Pir Cx differed from the left counterpart by an over-representation of mt genes (Supplementary Table S3, Figure 6B). The mt genome is a double-strand circular DNA with cellular copy numbers that vary between a few hundred and several thousand according to cell types. It contains 37 intronless genes that encode 13 peptides, 22 transfer RNAs, and 2 ribosomal RNAs involved in the translation of these peptides. Mitochondrial transcription generates polycistronic transcripts from which the mature mRNA, transfer RNA, and ribosomal RNA are derived, resulting in joint transcription of different mt genes [18,39]. Each of the 13 mt genes encoding peptides displayed significant or clear trends toward up-regulation in the right Ent-Pir Cx of 5G-exposed mice. This suggests that the 5G exposure resulted in transcription-sparing duplication or enhanced transcription of the mt-DNA. The mitochondrial genome encodes essential components of the OXPHOS, as illustrated by pathological consequences of mt gene mutations [40,41]. The OXPHOS comprises five protein complexes, four of which (complexes I to IV) ensure a stepwise series of transfer of electrons from reducing components to molecular oxygen. The process generates a transmembrane electrochemical gradient, which is the driving force for several mitochondrial functions, including the synthesis of ATP carried out by complex V (FoF1 ATP synthase) [17,42]. Reactive oxygen species (ROS) are common side products of oxidative phosphorylation, which predominantly originate from the activity of complexes I and III and can be a source of oxidative damage to DNA, proteins, and lipids [43,44].

Numerous studies have investigated the capacity of RF-EMF to promote ROS generation and tissue oxidative stress (reviewed in [45]). Increased ROS activity has been observed in the brain of rodents chronically exposed to RF-EMF at different frequencies (900, 1800, 2100, and 2450 MHz) used for 2–4G or WiFi communications, applying whole-body SAR values in the range from 0.6 × 10^−3^ to 0.9 W/kg [46,47,48,49,50,51]. In some studies, EMF-induced oxidative stress was associated with impaired spatial memory [51] or DNA damage [47,48,50]. With respect to the 3.5 GHz frequency band, Bektas et al. (2022) [52] found that repeated head exposures of adult rats (2 h/day for one month) to GSM-modulated 3.5 GHz resulted in enhanced levels of brain ROS that were assessed in whole brain homogenates, whereas SAR values were estimated at 323 mW/kg in the gray matter of the brain. Yang et al. (2022) [11] observed oxidative brain lesions marked by lipid peroxidation and swollen mitochondria when applying a continuous 3.5 GHz wave to adult guinea pigs for 3 days, with whole-body SAR values ≥ 2W/kg. Increased ROS production was also observed in cultured human astrocyte or glioma cell lines that were exposed to high-power 3.5 GHz pulses [53].

In line with the hypothesis that a 5G-modulated 3.5 GHz signal could promote ROS production, our study shows that exposure to the 5G-3.5 GHz signal increases the expression of mt genes encoding subunits of four of the five OXPHOS protein complexes (Supplementary Table S2), including the ROS-producing complexes I and III. However, the OXPHOS protein complexes involve the assembly of around 90 protein subunits, which have a dual mitochondrial and nuclear origin. Most of the subunits are encoded by nuclear genes and imported into the mitochondria [17]. Our RNA-seq analyses show no significant change in the expression of these nuclear genes. Furthermore, the cellular distribution of the mt gene response to the 5G-3.5 GHz signal is not known. This issue may have functional relevance, considering that the molecular assemblage of the OXPHOS complexes differs between neuronal and astroglial mitochondria, such that the production of ATP is more efficient in neurons, whereas ROS production is more prominent in astrocytes [54]. It remains to be determined whether the up-regulation of mt genes observed here reflects metabolism adaptations marked by enhanced production of ATP or oxidative stress, which could affect the right Ent-Pir Cx in the animal exposed to the 5G-3.5 GHz signal.

## 4. Conclusions and Limitations

Altogether, our results show that 1h of daily head exposure to a 5G-3.5 GHz signal over a six-week period does not alter emotional state and memory performance but triggers significant modification of expression in a limited set of genes, which can potentially affect glutamatergic synapses and mitochondrial activities. We acknowledge the limitations of our study. We cannot exclude that prolongations of the head exposures beyond 6 weeks could ultimately affect the emotional state or memory abilities of the exposed mice. While the classical OF tests used in our study did not show 5G-induced change in the animals’ behavior, we cannot rule out subtle alterations in emotional state that might be revealed by applying other behavioral tests, such as the elevated plus maze or emergence tests [55]. The experiments were carried out with male mice because of the impossibility of separately housing male and female mice during 5G exposure and behavioral testing. Further investigation will be required to evaluate whether female and male mice could be differentially affected by chronic exposure to 5G-3.5 GHz. In addition, our RNA-seq analyses were performed at a single time point, e.g., 24 h after the last exposure to the 5G-3.5 GHz signal. The kinetics and reversibility of the reported changes in gene expression are undetermined, and further studies are needed to assess whether and to what extent the changes in transcript levels translate into proteomic or functional alterations in mitochondria and in glutamatergic synapses. The 5G-triggered transcriptome modifications were observed in cortical areas where the average SAR levels range around 0.43 or 0.14 W/kg. These SAR levels may be considered in light of the European safety guidelines for human head exposures [56], which set the upper SAR limit to 2 W/kg. This value is higher than the corresponding SAR levels reached in the brain due to the energy absorption in the surrounding skull tissues. Recent dosimetric analyses of human exposures to downlink RF-EMF from base stations show intracortical SAR levels attributed to environmental 5G-3.5 GHz that are much lower than the values applied in our study, being less than 1 mW/kg [9]. Further investigations are needed to specify the levels of SAR reached in the human cerebral cortex when mobile phones emitting a 5G-3.5 GHz signal are held close to the ear of the mobile-phone user.

## 5. Materials and Methods

### 5.1. Subjects

Seven-week-old C57BL/6 J male mice (n = 32) were obtained from Charles River (L’Arbresle, France). They were housed in a humidity (50–55%) and temperature (22–24 °C)-controlled animal facility on a 12 h/12 h light/dark cycle (light on at 7:30 a.m.) with free access to food and water. After one week of familiarization with the animal facility, two groups of mice underwent a brief (30min) surgery under isoflurane anesthesia (1.5–2%) to secure a small plastic hook (0.25 g) to the skull bone with super-bond C&B (Sun Medical) and dental acrylic cement (Methax, Makevale Ltd., Herfordshire, UK). Metacam (0.1 mg/kg, i.p.) was administrated after the surgery to help the animal recover. This small hook allowed the animals to be held in restrained conditions during exposure or PSD exposure. Each mouse was weighed daily, and all mice included in this study gained weight after the initial surgery.

### 5.2. Exposure System and Exposure Protocol to 5G-3.5 GHz Signals

Head-only exposures to a 5G-3.5 GHz signal were performed in awake restrained mice. Each mouse was habituated to the exposure conditions by progressively increasing the time during which the mouse was head-fixed (from 15 min to 60 min over a week) in a red plastic tube (internal diameter: 32 mm) with the hook screwed to a small plastic post (see Figure 1A,B). Each day, a dipole antenna (SID3500, MVG, Plouzané, France) was positioned 5 mm from the animal’s head in a fixed/standardized position (Figure 1B) for one hour in the vicinity of the right temporal cortex.

The exposure system was similar to the one described in a previous study [35], replacing the frequency emitted by the radiofrequency generator to generate a 5G-3.5 GHz signal corresponding to 5G NR (release 15, Digital Standards SMBVB-K444; Rohde & Schwarz, Munich, Germany) with FDD duplexing, QPSK modulation, and a 100 MHz channel bandwidth. Briefly, a radiofrequency generator emitting a 5G-3.5 GHz electromagnetic field (SMBV100B, Rohde & Schwarz) was connected to a power amplifier (ZHL-4W-422+, Mini-Circuits, Brooklin, NY, USA), a circulator (Pasternack, PE83CR1005, Irvine, CA, USA), a bidirectional coupler (Mini-circuits, ZGBDC30-372HP+, Brooklin, NY, USA) and a four-way power divider (Mini-circuits ZB4PD-462W-N+, Brooklin, NY, USA), allowing potential simultaneous exposure of four animals. A power meter (E4417A and E9323A, EPM-P Series Power Meter, Agilent, Santa Clara, CA, USA) connected to the bidirectional coupler allowed continuous measurements and monitoring of incident and reflected powers within the setup.

Each exposed mouse was matched with a PSD-exposed mouse that was in head-fixed restrained conditions next to it (at about 30 cm, Figure 1A). An antenna was also placed 5 mm from the head of the PSD-exposed mouse, but this antenna was not connected to the 5G-3.5 GHz generator.

### 5.3. Dosimetry

Similar to previous studies [36,57,58], specific absorption rates (SARs) were determined numerically using a numerical mouse model with the finite difference time domain (FDTD) method [59,60,61] with a spatial resolution of 0.2 mm^3^. SARs were also determined experimentally in a homogeneous mouse model using a Luxtron probe for measurement of increases in temperature. In this case, SARs, expressed in W/kg, were calculated using the following equation: SAR = CΔT/Δt, with C being the calorific capacity in J/(kg·K), ΔT the temperature change in °K, and Δt the time in seconds. Numerically determined SAR values were compared with experimental SAR values obtained using homogenous models, especially in the equivalent mouse brain area. The difference between the numerical SAR determinations and the experimentally detected SAR values was less than 30%, as recommended by standard EMF exposure guidelines.

### 5.4. Behavioral Protocol

Twenty days after the beginning of the 5G exposure (Figure 2A), we assessed spontaneous locomotion, exploratory behavior, and potential anxiety in an OF arena, then long-term recognition memory using an object recognition task. These behavioral tasks were performed 10 min after the exposure or pseudo-exposure session. The testing procedure was similar to the one used in [62]. The protocol started with three habituation sessions to explore an OF (Figure 2B). The square OF (50 × 50 cm) had black sidewalls (35 cm high), and its floor was covered with 2 mm of sawdust. A camera connected to a video-tracking system (ANY-maze^TM^ Video Tracking System, version 7.3, Stoelting, Wood Dale, IL, USA) was placed above the OF to record the mice’s activity. Experiments were undertaken under homogeneous dim illumination (<50 lux). On days 1–3, each mouse was allowed to explore the empty OF for 20 min (day 1) or 10 min (days 2 and 3). During these three sessions, the total distance traveled, number of rearings, and percentage of distance traveled in the peripheral area (5 cm from the walls) were recorded to evaluate locomotion, exploration, and potential anxiety revealed by thigmotaxis (tendency of an anxious mouse to remain close to walls). To assess novelty-seeking behavior, on day 4, two identical rectangular plastic objects were placed in the center zone, and the mice could freely explore them for 10 min. The time exploring the objects, as well as the distances traveled and thigmotaxis, were recorded. Object exploration was defined as the mouse touching the object with its nose or sniffing it with a distance between the nose and the object of less than 2 cm. In the following days (days 5–8), mice were tested in two successive experiments: the novel object recognition task (NOR) to assess object recognition memory and the object location task (OL) to assess spatial memory (Figure 2B). The order of these experiments was counterbalanced among individuals and groups (control mice, exposed mice, pseudo-exposed mice). Each experiment consisted of an acquisition session followed by a retention test 24 h later (Figure 2B). Thus, on day 5 or 7, three different objects were placed in the OF and could be freely explored during three consecutive trials of 5 min with a 5 min inter-trial interval (ITI). The objects were plastic toys of different colors and shapes (3–6 cm diameter, 3–6 cm high). The objects and their spatial arrangement in the test box were chosen in a pseudorandom order and were counterbalanced between mice. Twenty-four hours later (days 6 and 8), a memory test (one trial of 5 min) was performed (Figure 2B). For these tests, because mice are spontaneously attracted by novelty, one object explored during the previous acquisition session was replaced with a novel object to assess object recognition memory, and one of the three objects explored during the previous acquisition session was displaced to a novel location to assess object location memory (days 6 and 8, Figure 2B). For each session (acquisition and retention), the time spent exploring each object was analyzed, as well as the total object exploration and total distance traveled. For each subject, retention performance was expressed as the percentage of time spent exploring the novel/displaced object over the total object exploration time during the 5 min session.

### 5.5. Statistical Analyses

For all behavioral statistical analyses, GraphPad Prism (version 8.0.2) was used. The data are presented as the group means ± SEM. For each parameter collected during the behavioral tests, the normality of the distribution and the homogeneity of variance were checked before running the statistical analyses (respectively, the Shapiro–Wilk test and Levene’s test). For all analyses, no outliers were identified (ROUT method (Q = 1%)). Differences among groups were assessed by one-way analysis of variance (ANOVA) or two-way ANOVA for multifactorial analyses. As appropriate, Bonferroni’s post hoc tests were employed to determine group differences. For the memory test, one sample *t*-test was used to compare the exploration of the displaced/novel object to the chance level (33.33%). The significance level was set at *p* < 0.05.

### 5.6. Tissue Preparation for Transcriptome Analyses

Mice were killed by decapitation under isoflurane anesthesia 24 h after the last behavioral test. The brains were quickly removed from the skulls and frozen. Tissue samples were extracted from a brain region covering the adjacent area of the entorhinal and the piriform cortex (Ent-Pir Cx) of the right and left hemispheres in 100 µm-thick coronal sections cut on a cryostat and distributed over a cortical region starting 9 mm caudal to the anterior part of the olfactory bulb and extending over 1 mm in the rostrocaudal axis. Tissues were stored frozen at −80 °C until use.

### 5.7. RNA-Seq and Data Analysis

Total RNA was extracted from the Ent-Pir Cx collected from the right and left hemispheres of PSD-exposed (n = 8) or 5G-exposed (n = 7) mice using an RNeasy^®^ Micro kit (QIAGEN, Redwood City, CA, USA). The quality of the RNA was confirmed on an Agilent TapeStation (RIN > 8). RNA-seq libraries were prepared using an Illumina Stranded mRNA Prep kit (Illumina) and sequenced with the NovaSeq 6000 platform (Illumina, Inc., San Diego, CA,USA) (2 × 87 million reads with 100 nucleotides length per sample).

The quality of the raw data was evaluated with FastQC [63]. Poor-quality sequences and adapters were trimmed or removed with the fastp tool with default parameters to retain only good-quality paired reads [64]. The Illumina DRAGEN bio-IT Platform (v3.10.4) was used for mapping on an mm10 reference genome and quantification with the gencode vM25 annotation gtf file. Library orientation, library composition, and coverage along transcripts were checked with Picard tools. The following analyses were conducted with R software (v4.2.1) Data were normalized with the DESeq2 (v1.34.0) bioconductor package [65] prior to differential analysis with the glm framework likelihood ratio test from the DESeq2 workflow. Multiple hypothesis-adjusted *p*-values were calculated with the Benjamini–Hochberg procedure to control FDR. Results were considered statistically significant for FDR-adjusted *p*-values ≤ 0.05, and the fold-change (FC) threshold was set to 1.2 to determine DEG. Clusterings were performed using the “dist” and “hclust” functions in R, using the Euclidean distance and Ward agglomeration methods. Finally, over-representation enrichment analysis was conducted with the DAVID Functional Annotation Tool [66,67]. GO terms were considered enriched if fold enrichment ≥ 2.0, FDR ≤ 0.05, and a minimum number of regulated genes in the pathway/term ≥ 2.0.

## Data Availability

The RNA-seq datasets generated and analyzed during the current study are available in the Gene Expression Omnibus (GEO)–NCBI database repository. Accession number: GSE289315.

## Supplementary Materials

The following supporting information can be downloaded at: https://www.mdpi.com/article/10.3390/ijms26062459/s1.

## Abbreviations

The following abbreviations are used in this manuscript:

ANOVA	Analysis of Variance
CTRL	Control
CNS	Central Nervous System
DEG	Differentially Expressed Genes
EMF	Electromagnetic Fields
Ent-Pir Cx	Entorhinal and Piriform Cortex
EXP	5G-exposed
FC	Fold-Change
FDR	False Discovery Rate
GO	Gene Ontology
G	Generation
ITI	Inter Trial Interval
mt	Mitochondrial
NOR	Novel Object Recognition
OF	Open Field
OL	Object Location
OXPHOS	Oxidative Phosphorylation System
PSD	Pseudo
RF	Radiofrequency
SAR	Specific Absorption Rate
