# Supplementary material for: Repeated Head Exposures to a 5G-3.5 GHz Signal Do Not Alter Behavior but Modify Intracortical Gene Expression in Adult Male Mice
Source: Int J Mol Sci. 2025 Mar 10;26(6):2459. doi: 10.3390/ijms26062459 (PMC11941837; doi:10.3390/ijms26062459)
Supplement: Supplementary file 1 [file ijms-26-02459-s001.zip › ijms-3456922-supplementary.pdf]

Supplementary Figure S1

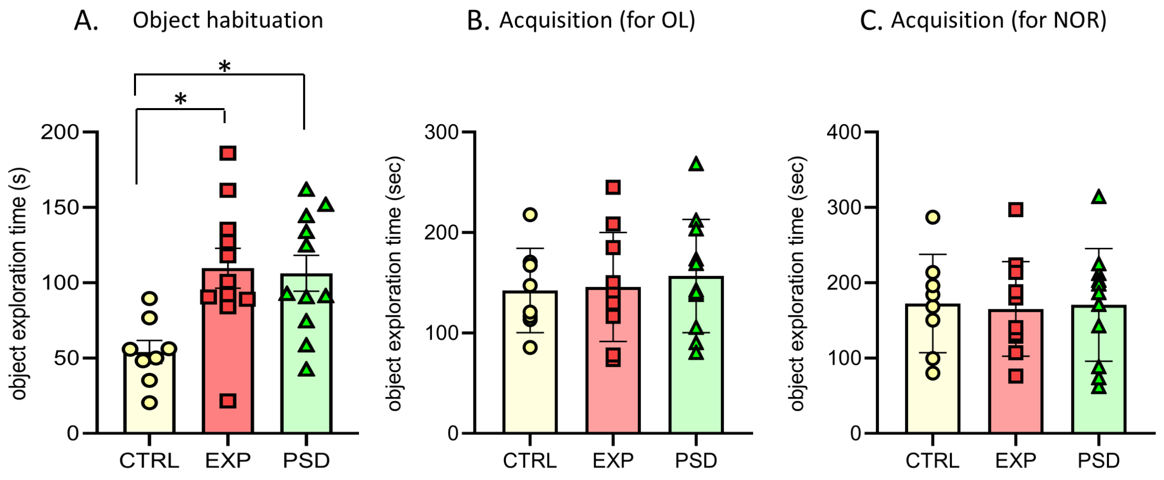

Supplementary Figure S1. (A) Object time exploration during the object habituation session (day 4) for CTRL (n=8), EXP (5G-exposed) (n=11) and PSD (n=11) mice. One-way ANOVA, P<0.05- Bonferroni's multiple comparisons test; CTRL vs EXP P<0.05; CTRL vs PSD: P<0.05; EXP vs PSD: ns (B-C) Object time exploration during the acquisition sessions (days 5 or 7) for CTRL (n=8), EXP (n=10/11) and PSD (n=11) mice. One-way ANOVA, ns. CTRL: control mice; EXP: 5G-exposed mice; PSD: Pseudo-exposed mice. * indicates significant difference. ns: non significant.

Supplementary Table S1

	CTRL	EXP	PSD	ANOVA
**2nd session - OF**				
Travelled distance	57.14 ± 3.47	57.75 ± 1.98	54.81 ± 2.76	$F(2,28)=0.36$; $P=0.69$
Numbers of rearings	79.13 ± 7.06	93.83 ± 5.99	88.09 ± 6.72	$F(2,28)=1.16$; $P=0.32$
Travelled distance in center (%)	46.15 ± 2.28	50.17 ± 2.08	50.11 ± 2.3	$F(2,28)=0.91$; $P=0.41$
**3rd session - OF**				
Travelled distance	52.13 ± 1.9	54.74 ± 2.43	58.4 ± 3.27	$F(2,28)=1.22$; $P=0.30$
Numbers of rearings	94.5 ± 7.91	97.42 ± 5.21	100.2 ± 6.39	$F(2,28)=0.18$; $P=0.83$
Travelled distance in center (%)	45.14 ± 3	43.44 ± 1.65	50.28 ± 2.82	$F(2,28)=2.26$; $P=0.12$

Supplementary Table S1: Behavioral parameters observed during the 2nd and 3rd session in openfield (OF) and statistical results (factorial ANOVA). CTRL: control mice; EXP: 5G-exposed mice; PSD: Pseudo-exposed mice.

Supplementary Table S2 - Genes Differentially Expressed in the Right and the Left Ent-Pir CX in Pseudo-exposed Animals

Gene symbol	Gene Name	Expression Right versus Left Ent-Pir CX	Fold-change	adjusted p value
Ankfn1	ankyrin-repeat and fibronectin type III domain containing 1	Down	2,001998642	0,042075965
Gnb4	guanine nucleotide binding protein (G protein), beta 4	Down	1,44379541	0,042075965
Igfbp6	insulin-like growth factor binding protein 6	Down	1,703388817	0,042075965
Ptgs2	prostaglandin-endoperoxide synthase 2	Down	1,849700874	0,042075965
Smoc2	SPARC related modular calcium binding 2	Down	1,717193717	0,042075965

Supplementary Table S3 - Genes Differentially Expressed in the Right Ent- Pir CX of 5G/3.5GHz-exposed versus Pseudo-exposed Mice

Gene symbol	Gene Name	Regulation	Fold Change	Adjusted P Value
Tmem215	transmembrane protein 215	Up	2,10123775	0,035256269
mt-Atp8	mitochondrially encoded ATP synthase membrane subunit 8	Up	1,823962922	0,047680182
Tafa1	TAFA chemokine like family member 1	Up	1,72145799	0,013185702
Nbl1	NBL1, DAN family BMP antagonist	Up	1,698791208	0,00613382
Colec12	collectin sub-family member 12	Up	1,657607827	0,019651156
mt-Nd5	mitochondrially encoded NADH:ubiquinone oxidoreductase core subunit 5	Up	1,641162935	0,00815959
Commd1b	COMM domain containing 1B	Up	1,62732475	0,035256269
E130008D07Rik	RIKEN cDNA E130008D07 gene	Up	1,611660195	0,000108869
Sult1a1	sulfotransferase family 1A, phenol-preferring, member 1	Up	1,550381317	0,036501427
mt-Nd4	mitochondrially encoded NADH:ubiquinone oxidoreductase core subunit 4	Up	1,53136465	0,006650248
Ddr2	discoidin domain receptor family, member 2	Up	1,524039552	0,042791558
1700001L05Rik	RIKEN cDNA 1700001L05 gene	Up	1,517800023	0,035256269
mt-Nd2	mitochondrially encoded NADH:ubiquinone oxidoreductase core subunit 2	Up	1,514910352	0,010836328
mt-Atp6	mitochondrially encoded ATP synthase membrane subunit 6	Up	1,500275142	0,018944593
Mylk	myosin, light polypeptide kinase	Up	1,495777779	0,042791558
Oxtr	oxytocin receptor	Up	1,481816164	0,035256269
mt-Nd1	mitochondrially encoded NADH:ubiquinone oxidoreductase core subunit 1	Up	1,479149681	0,00613382
Dgkb	diacylglycerol kinase, beta	Up	1,478827252	0,028157361
Cyp4v3	cytochrome P450, family 4, subfamily v, polypeptide 3	Up	1,477909291	0,028715495
Bicc1	BicC family RNA binding protein 1	Up	1,467216077	0,041515547
Lmo7	LIM domain only 7	Up	1,46641479	0,042791558
Camk2a	calcium/calmodulin-dependent protein kinase II alpha	Up	1,446929583	0,042533337
mt-Cytb	mitochondrially encoded cytochrome b	Up	1,425845255	0,00613382
Tusc1	tumor suppressor candidate 1	Up	1,424969226	0,038636782
Necab2	N-terminal EF-hand calcium binding protein 2	Up	1,411584928	0,001101849
mt-Co1	mitochondrially encoded cytochrome C oxidase 1	Up	1,405973494	0,013286464
Luzp2	leucine zipper protein 2	Up	1,386123361	0,027510233
mt-Nd3	mitochondrially encoded NADH:ubiquinone oxidoreductase core subunit 3	Up	1,386086987	0,035256269
Cald1	caldesmon 1	Up	1,384870919	0,039715884
Tgfbr3	transforming growth factor, beta receptor III	Up	1,37615325	0,019496624
mt-Co2	mitochondrially encoded cytochrome C oxidase 2	Up	1,371095799	0,018126119
B130046B21Rik	RIKEN cDNA B130046B21 gene	Up	1,336930297	0,042791558
Neurl1a	neuralized E3 ubiquitin protein ligase 1A	Up	1,336818834	0,00613382
Ddn	dendrin	Up	1,316029909	0,017775078
Aqp11	aquaporin 11	Up	1,315881515	0,042791558
Slc30a10	solute carrier family 30, member 10	Up	1,275503312	0,042791558
F3	coagulation factor III	Up	1,269633429	0,042791558
Pts	6-pyruvoyl-tetrahydropterin synthase	Up	1,230450551	0,00815959
Cryl1	crystallin, lambda 1	Up	1,219596552	0,002607393
Phactr3	phosphatase and actin regulator 3	Up	1,21875568	0,006650248
Narf	nuclear prelamin A recognition factor	Down	1,204361344	0,042791558
Plekha6	pleckstrin homology domain containing, family A member 6	Down	1,209090277	0,042791558
Flywch1	FLYWCH-type zinc finger 1	Down	1,211330354	0,000864552
Zfp14	zinc finger protein 14	Down	1,225111318	0,014383596
Frmd5	FERM domain containing 5	Down	1,23726309	0,042791558
Kcnk3	potassium channel, subfamily K, member 3	Down	1,240114756	0,00613382
Relt	RELT tumor necrosis factor receptor	Down	1,240379227	0,034746623
Mis12	MIS12 kinetochore complex component	Down	1,243419411	0,019167415
Srd5a1	steroid 5 alpha-reductase 1	Down	1,249641163	0,016316321
Ckmt1	creatine kinase, mitochondrial 1, ubiquitous	Down	1,277217645	0,040529978
Dnm3	dynamin 3	Down	1,27842835	0,042791558
Dpy19l1	dpy-19-like 1 (C. elegans)	Down	1,279792466	0,035256269
Efhd2	EF hand domain containing 2	Down	1,286468075	0,026586843
Plk3	polo like kinase 3	Down	1,302618551	0,018944593
B3galt2	UDP-Gal:betaGlcNAc beta 1,3-galactosyltransferase, polypeptide 2	Down	1,32041819	0,037667774
Rcan2	regulator of calcineurin 2	Down	1,331724382	0,033980702
Kcnip1	Kv channel-interacting protein 1	Down	1,343305636	0,019167415
Cdc42ep2	CDC42 effector protein (Rho GTPase binding) 2	Down	1,352317815	0,040529978
Astn2	astrotactin 2	Down	1,367873324	0,035256269
Sema5b	sema domain, seven thrombospondin repeats (type 1 and type 1-like), transme	Down	1,398864928	0,019651156
Ksr1	kinase suppressor of ras 1	Down	1,441313472	0,034419973
Zmat4	zinc finger, matrin type 4	Down	1,449308068	0,000108869
Adgra1	adhesion G protein-coupled receptor A1	Down	1,454428578	0,02908442
1110032F04Rik	RIKEN cDNA 1110032F04 gene	Down	1,510996271	0,024892683
Lrrc55	leucine rich repeat containing 55	Down	1,515679193	0,003491699
Kcnab3	potassium voltage-gated channel, shaker-related subfamily, beta member 3	Down	1,518304789	0,000365708
Cntnap4	contactin associated protein-like 4	Down	1,586745711	0,042791558
Fosl2	fos-like antigen 2	Down	1,594463874	0,017775078
Plcb4	phospholipase C, beta 4	Down	1,603384762	6,83427E-05
Chrm2	cholinergic receptor, muscarinic 2, cardiac	Down	1,605751483	0,000739057
1700019D03Rik	RIKEN cDNA 1700019D03 gene	Down	1,642167322	0,00613382
Cdh6	cadherin 6	Down	1,752194086	0,000997876
Fezf2	Fez family zinc finger 2	Down	1,854000037	0,00613382
Cbln2	cerebellin 2 precursor protein	Down	1,956795077	0,001943559
Pou3f1	POU domain, class 3, transcription factor 1	Down	2,104583328	0,00613382
Chrna5	cholinergic receptor, nicotinic, alpha polypeptide 5	Down	2,114319789	0,035256269
Syt2	synaptotagmin II	Down	2,280330988	0,035256269

Supplementary Table S4 - Genes Differentially Expressed in the Left Ent- Pir CX of 5G/3.5GHz- exposed versus Pseudo-exposed Mice

Gene Symbol	Gene Name	Regulation	Fold-Change	Adjusted P value
Hydin	HYDIN, axonemal central pair apparatus protein	Up	2,172211709	0,017208957
Tcfl5	transcription factor-like 5 (basic helix-loop-helix)	Up	1,881394456	0,043223092
Emid1	EMI domain containing 1	Up	1,731889225	0,037644192
Prcd	photoreceptor disc component	Up	1,686086308	0,03838455
Gm20033	predicted gene,20033	Up	1,599096456	0,021280321
Gm19531	predicted gene, 19531	Up	1,566355405	0,003170859
Fndc1	fibronectin type III domain containing 1	Up	1,543010288	0,02225779
Crb2	crumbs family member 2	Up	1,450553024	0,04478391
Rreb1	ras responsive element binding protein 1	Up	1,432848572	0,000184779
Hpcal1	hippocalcin-like 1	Up	1,429724219	0,021759428
4933439C10Ri	RIKEN cDNA 4933439C10 gene	Up	1,391404724	0,024485058
Rxrg	retinoid X receptor gamma	Up	1,387955969	0,04478391
Csf2ra	colony stimulating factor 2 receptor, alpha, low-affinity (gr	Up	1,354494942	0,023343479
Gm10076	ribosomal protein L41 pseudogene	Up	1,342907715	0,025497401
Marcksl1	MARCKS-like 1	Up	1,326727892	0,022304274
Necab2	N-terminal EF-hand calcium binding protein 2	Up	1,318424142	0,041436256
Znhit2	zinc finger, HIT domain containing 2	Up	1,302204002	0,000858727
2900079G21Ri	RIKEN cDNA 2900079G21 gene	Up	1,29588456	0,037983959
Nrros	negative regulator of reactive oxygen species	Up	1,283615708	0,043635616
Slc9a5	solute carrier family 9 (sodium/hydrogen exchanger), mem	Up	1,276785779	0,043555139
Spint2	serine protease inhibitor, Kunitz type 2	Up	1,26960991	0,015886463
Smpd4	sphingomyelin phosphodiesterase 4	Up	1,26216437	0,039904621
Ppan	peter pan homolog	Up	1,25592274	0,022253586
Romo1	reactive oxygen species modulator 1	Up	1,249512356	0,019545498
Ccdc86	coiled-coil domain containing 86	Up	1,247139085	0,007263344
Mapk3	mitogen-activated protein kinase 3	Up	1,224174918	0,024703839
Fcho1	FCH domain only 1	Up	1,221178822	0,048607287
Ly6h	lymphocyte antigen 6 complex, locus H	Up	1,220949679	0,024485058
Clba1	clathrin binding box of aftiphilin containing 1	Up	1,217249232	0,022304274
Fjx1	four jointed box 1	Up	1,206222912	0,031009214
Rb1cc1	RB1-inducible coiled-coil 1	Down	1,202395392	0,043223092
Pik3ca	phosphatidylinositol-4,5-bisphosphate 3-kinase catalytic s	Down	1,20710086	0,007263344
Ina	internexin neuronal intermediate filament protein, alpha	Down	1,209377858	0,016682578
Synj2bp	synaptojanin 2 binding protein	Down	1,213839042	0,037983959
Pou3f3	POU domain, class 3, transcription factor 3	Down	1,21485427	0,024301812
Foxn3	forkhead box N3	Down	1,219177411	0,018717556
Dixdc1	DIX domain containing 1	Down	1,229569255	0,013233532
Sacm1l	SAC1 suppressor of actin mutations 1-like (yeast)	Down	1,230607801	1,03167E-06
Slc35g2	solute carrier family 35, member G2	Down	1,231926857	0,015003021
Abcd2	ATP-binding cassette, sub-family D (ALD), member 2	Down	1,24440556	0,028487069
Bcat1	branched chain aminotransferase 1, cytosolic	Down	1,249249706	0,033303483
Ppargc1a	peroxisome proliferative activated receptor, gamma, coac	Down	1,250246951	0,000238214
Hspa4l	heat shock protein 4 like	Down	1,259530222	0,031767419
Kcna2	potassium voltage-gated channel, shaker-related subfamil	Down	1,259840487	0,04391815
Slc30a4	solute carrier family 30 (zinc transporter), member 4	Down	1,261104909	0,009843548
Slc38a2	solute carrier family 38, member 2	Down	1,262395672	0,007133097
Mpp6	m-phase phosphoprotein 6	Down	1,263107253	0,019545498
Slc24a2	solute carrier family 24 (sodium/potassium/calcium excha	Down	1,273349168	0,021206078
Oprd1	opioid receptor, delta 1	Down	1,283152636	0,036056021
Ephb2	Eph receptor B2	Down	1,28874879	0,04478391
Cacna1i	calcium channel, voltage-dependent, alpha 1I subunit	Down	1,288967143	9,10616E-05
Zfp970	zinc finger protein 970	Down	1,296740165	0,003170859
Gm4631	predicted gene 4631	Down	1,302024897	0,015886463
Clstn2	calsyntenin 2	Down	1,305322599	0,015886463
Zfp874b	zinc finger protein 874b	Down	1,312915693	0,032435982
Luzp1	leucine zipper protein 1	Down	1,315208244	5,87158E-09
Rcan2	regulator of calcineurin 2	Down	1,341489279	0,024841551
Dkk3	dickkopf WNT signaling pathway inhibitor 3	Down	1,348012288	0,024301812
Hlf	hepatic leukemia factor	Down	1,349432506	0,007263344
Adamts1	a disintegrin-like and metallopeptidase (reprolysin type) w	Down	1,371199666	0,015886463
Fndc5	fibronectin type III domain containing 5	Down	1,394396686	0,000380597
Scn1a	sodium channel, voltage-gated, type I, alpha	Down	1,449732114	0,000346123
Zmat4	zinc finger, matrin type 4	Down	1,477661447	0,006388562
Vamp1	vesicle-associated membrane protein 1	Down	1,507089657	0,034053329
Gm14403	predicted gene 14403	Down	1,511178465	0,003410025
Plxdc1	plexin domain containing 1	Down	1,524801317	0,003550648
Stac2	SH3 and cysteine rich domain 2	Down	1,539397661	0,04478391
Trim59	tripartite motif-containing 59	Down	1,543670584	0,035867429
Myrf	myelin regulatory factor	Down	1,546300211	0,04391815
Cd34	CD34 antigen	Down	1,549170763	0,000238214
Piga	phosphatidylinositol glycan anchor biosynthesis, class A	Down	1,560069365	0,000289933
Plcb4	phospholipase C, beta 4	Down	1,582364854	0,01560624
Pcdhb4	protocadherin beta 4	Down	1,621115048	0,049399472
Ugt8a	UDP galactosyltransferase 8A	Down	1,671397595	0,043904701
Cbln2	cerebellin 2 precursor protein	Down	1,693082121	0,033919813
Pvalb	parvalbumin	Down	1,695706187	0,024301812
Serpinb1a	serine (or cysteine) peptidase inhibitor, clade B, member 1	Down	1,751115561	0,036357515
Lrrc55	leucine rich repeat containing 55	Down	1,755707019	0,000380597
Chrm2	cholinergic receptor, muscarinic 2, cardiac	Down	1,881383141	0,000915282
Ifit1	interferon-induced protein with tetratricopeptide repeats	Down	2,05813761	3,48491E-05
Rab37	RAB37, member RAS oncogene family	Down	2,764226055	0,015886463
Scn4b	sodium channel, type IV, beta	Down	2,862554279	0,04478391
Syt2	synaptotagmin II	Down	3,309050302	0,000261677
Lct	lactase	Down	3,50795283	0,031865617
